# Opening a new window of interventional endoscopic ultrasound using a 22-G needle with a novel dedicated dilation device

**DOI:** 10.1055/a-2489-8334

**Published:** 2024-12-10

**Authors:** Takeshi Ogura

**Affiliations:** 1 Endoscopy Center, Osaka Medical and Pharmaceutical University Hospital, Osaka, Japan

Interventional endoscopic ultrasound (EUS) including hepaticogastrostomy (HGS) or pancreatic duct drainage is now widely performed as an alternative method when endoscopic retrograde cholangiopancreatography fails. However, intrahepatic bile duct or main pancreatic duct puncture using a 19-G needle might be challenging, especially in nonexpert hands. In such cases, puncture using a 22-G needle might reduce the technical difficulty. However, because only a 0.018-inch guidewire can be used under 22-G needle guidance, device exchange may be unstable, and tract dilation may also be challenging 1 . Recently, a double guidewire technique has been reported for matching the axis between the device insertion vector and the tract and obtaining a stable echoendoscope 2 .

When performing the double guidewire technique, a 0.018-inch guidewire is deployed and then several device exchanges are needed. In this situation, bile leakage from the fistula can occur. To overcome these issues, we developed a novel dilation device (Meissa, type OT; Japan Life Line, Tokyo, Japan) ( Fig. 1 ). The tip of this device is 2.3 Fr, and the maximum diameter is 7.4 Fr. In addition, between the tip and 2 cm, a side hole is provided. Contrast medium injection, aspiration of bile juice, and 0.025-inch guidewire insertion can be performed through this hole. Therefore, if this device is used, the double guidewire technique can be performed without additional device exchange under a 0018-inch guidewire. Herein, we described a case of EUS-HGS using a 22-G needle with a novel dilation device.

**Fig. 1:**
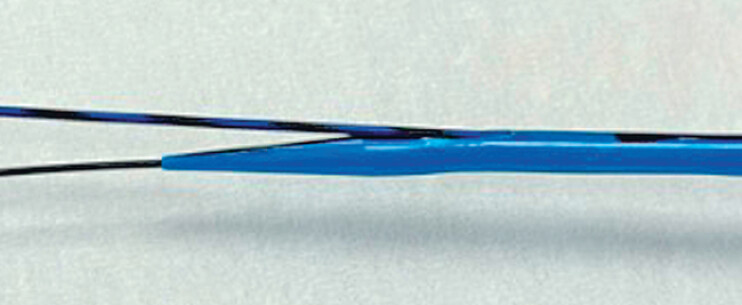
A novel dilation device (Meissa, type OT, Japan Life Line, Tokyo, Japan). The tip of this device is 2.3 Fr, and the maximum diameter is 7.4 Fr. In addition, between the tip and 2 cm, a side hole is provided. Contrast medium injection, aspiration of bile juice, and 0.025-inch guidewire insertion can be performed.

A 77-year-old man was admitted to our hospital due to obstructive jaundice caused by cancer of the head of the pancreas. EUS-HGS was attempted because of duodenal obstruction. The intrahepatic bile duct was successfully and easily punctured using a 22-G needle, and contrast medium was injected. Then, a 0.018-inch guidewire was deployed ( Fig. 2 **a** ). Next, the novel dilation device was smoothly and easily inserted into the biliary tract ( Fig. 2 **b** ). After contrast medium injection from the side hole to evaluate the obstruction site, a 0.025-inch guidewire was deployed through this dilation device. By doing so, the double guidewire technique was easily performed ( Fig. 2 **c** ). Finally, an 8.5-Fr stent delivery system was inserted into the biliary tract without additional tract dilation and was successfully deployed from the intrahepatic bile duct to the stomach ( Fig. 2 **d** , Video 1 ). No procedure-related adverse events were observed, and obstructive jaundice was resolved.

**Fig. 2:**
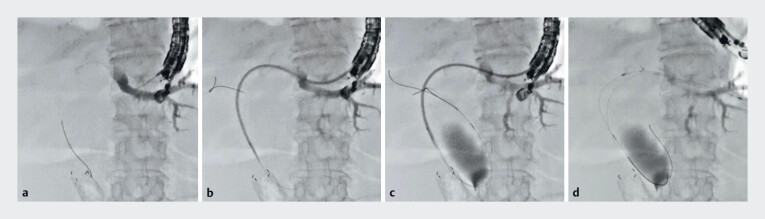
Use of the novel dilation device with the double guidewire technique. **a** After successful bile duct puncture using the 22-G needle, a 0.018-inch guidewire was deployed. **b** The novel dilation device was smoothly and easily inserted into the biliary tract. **c** A 0.025-inch guidewire was deployed through the side hole on the novel dilation device. **d** An 8.5-Fr stent delivery system was inserted into the biliary tract without additional tract dilation and was successfully deployed from the intrahepatic bile duct to the stomach.

A double guidewire technique using a novel dilation device.Video 1

In conclusion, this novel dilation device opens a new window of interventional EUS using a 22-G needle.

Endoscopy_UCTN_Code_TTT_1AS_2AH

## Notes

Endoscopy E-Videos https://eref.thieme.de/e-videos
*E-Videos* is an open access online section of the journal *Endoscopy* , reporting on interesting cases and new techniques in gastroenterological endoscopy. All papers include a high-quality video and are published with a Creative Commons CC-BY license. Endoscopy E-Videos qualify for HINARI discounts and waivers and eligibility is automatically checked during the submission process. We grant 100% waivers to articles whose corresponding authors are based in Group A countries and 50% waivers to those who are based in Group B countries as classified by Research4Life (see: https://www.research4life.org/access/eligibility/ ). This section has its own submission website at https://mc.manuscriptcentral.com/e-videos .
